# Factors associated with internalizing or somatic symptoms in a cross-sectional study of school children in grades 1-10

**DOI:** 10.1186/1753-2000-4-33

**Published:** 2010-12-17

**Authors:** Audhild Løhre, Stian Lydersen, Lars J Vatten

**Affiliations:** 1 Department of Public Health, Faculty of Medicine, Norwegian University of Science and Technology, Trondheim, Norway; 2 The Central Norway Regional Health Authority (RHA), Norway; 3 Unit for Applied Clinical Research, Department of Cancer Research and Molecular Medicine, Faculty of Medicine, Norwegian University of Science and Technology, Trondheim, Norway

## Abstract

**Abstract:**

**Background**

School related factors that may contribute to children's subjective health have not been extensively studied. We assessed whether factors assumed to promote health and factors assumed to have adverse effects were associated with self-reported internalizing or somatic symptoms.

**Methods**

In a cross-sectional study, 230 boys and 189 girls in grades 1-10 from five schools responded to the same set of questions. Proportional odds logistic regression was used to assess associations of school related factors with the prevalence of sadness, anxiety, stomach ache, and headache.

**Results:**

In multivariable analyses, perceived loneliness showed strong and positive associations with sadness (odds ratio, 1.94, 95% CI 1.42 to 2.64), anxiety (odds ratio, 1.78, 95% CI 1.31 to 2.42), and headache (odds ratio, 1.47, 95% CI 1.10 to 1.96), with consistently stronger associations for girls than boys. Among assumed health promoting factors, receiving necessary help from teachers was associated with lower prevalence of stomach ache in girls (odds ratio, 0.51, 95% CI 0.30 to 0.87).

**Conclusions**

These findings suggest that perceived loneliness may be strongly related to both internalizing and somatic symptoms among school children, and for girls, the associations of loneliness appear to be particularly strong.

## Background

Children's perceived health status influences their daily life [1,2], and childhood health is also a powerful predictor for health in adulthood [3,4]. Health complaints are typically classified as either emotional or somatic, and a combination of these types of symptoms is not uncommon [5-10].

Anxiety and depression are the most common emotional problems, and appear to be more prevalent among girls, with fairly high co-morbidity (20-50%) [11]. Anxiety tends to predate depression [6,9], and the prevalence may range from 6% to 18% in childhood and adolescence [11]. Depressive disorders are rare among young children, but in adolescence the prevalence may be as high as 8% [11]. The results of long term follow-up studies suggest that early emotional symptoms may predict higher risk of mental and physical disease in middle age [12-14].

Headache and stomach pain are the most prevalent physical complaints at a young age [15]. Before elementary school, children rarely complain about headache [16], but the prevalence increases with age [10,17,18]. Around puberty, about 15% may report frequent or severe headache, and more than half of the students in high school may report less frequent episodes of headache [17]. Before puberty, the prevalence of reported headache seems to be higher in boys than girls, but after puberty, the prevalence appears to be higher among girls [17,18].

Stomach pain appears to be more frequent among younger than older children [16,19,20]. Recurrence of abdominal pain may range from 10-45% [21], and in adolescence (11-15 years), the total prevalence of self-reported episodes of stomach pain is around 50%, and the estimates are higher for girls than boys [20,22]. Perceived abdominal pain in childhood has been associated with higher risk of both physical and mental disorders later in life [23,24].

In school, both circumstances in class and during recess may be important for the children's health and wellbeing. Learning disabilities, low academic achievement or emotional distress may be associated with poorer health [22,25-28]. Victimization caused by bullying, as well as perceived loneliness, have also been associated with adverse health effects, both in the short and long term [29-31]. Further, experiencing caring teachers and belonging to school have been related to good health and wellbeing [32], and negatively associated with emotional distress and risky behaviour [33,34]. There is also evidence to suggest that connectedness to school may be associated with better health in the long term and less risky behaviour [35-37].

In the present study of more than 400 school children, we collected information on self-reported sadness and anxiety, and headache and stomach ache. The aim was to assess whether factors assumed to influence health status, either negatively or positively, were associated with the prevalence of the four symptoms.

## Methods

### Participants and procedure

This study is based on a convenience sample of children from five schools in Møre and Romsdal County, Norway, who participated in a project that was organized by the schools. The headmasters agreed to participate in two cross sectional surveys that were set two years apart. The headmasters' decision was approved by each School's Collaborative Committee (sanctioned by law, and including representatives for teachers, parents and children). In the present study, data were used from the first survey that was carried out from May to June 2002.

Three schools had grades from 1 to 7, and two schools had grades from 1 to 10. Altogether 423 children were invited, and included all children from four of the schools and children in grades 7-10 from the fifth school. The children were between seven and 16 years of age at attendance. One child moved before the data collection started, and three children were on sick leave during the study period. Thus, 419 (99%) children were included in the analyses.

Parents were informed about the survey in the context of a school meeting that indicated the start of the project. Information letters signed by the headmaster and by the principal investigator (AL) were sent to all parents, describing the aims of the survey, and emphasising that participation was voluntary, and that the collected information was confidential. Children/parents who did not want to participate were asked to notify their main teacher or headmaster. In each class, teachers informed the children in greater detail about the survey.

In this study, we applied a questionnaire that has been described in more detail elsewhere [38]. The reliability of the questionnaire was tested in another material gathered from children in grades 3, 6, and 9. Of 179 eligible children, the questionnaire was completed by 154 (86%) children two times with three weeks apart. The test-retest reliability for the 49 ordinal questions was acceptable with 82% of the Spearman's rho coefficients ranging between 0.45 and 0.64 (mean rho = 0.55), and all p-values < 0.001. With regard to the 15 variables used in the present study correlations varied from 0.46 to 0.71.

The data collection of the present study was administered by school nurses and headmasters. Instead of letting all children fill in the questionnaire themselves, 180 children in grades 1-4, 53 children in grades 5-7, and three children in grades 8-10 were interviewed by trained school nurses who used the questionnaire as a guide. Under the instruction of the school nurse or a trained teacher the remaining 183 children completed the questionnaires themselves during a lesson that was allocated to this task.

### Measures

Children's health symptoms were measured by four questions: "Lately, how often have you felt: 1) sadness; 2) anxiety; 3) stomach ache; or 4) headache?" Each question had five response options; never (1), seldom, sometimes, often, and always (5). Sadness and anxiety were denoted internalizing symptoms, stomach ache and headache were denoted somatic symptoms.

**Figure 1:**
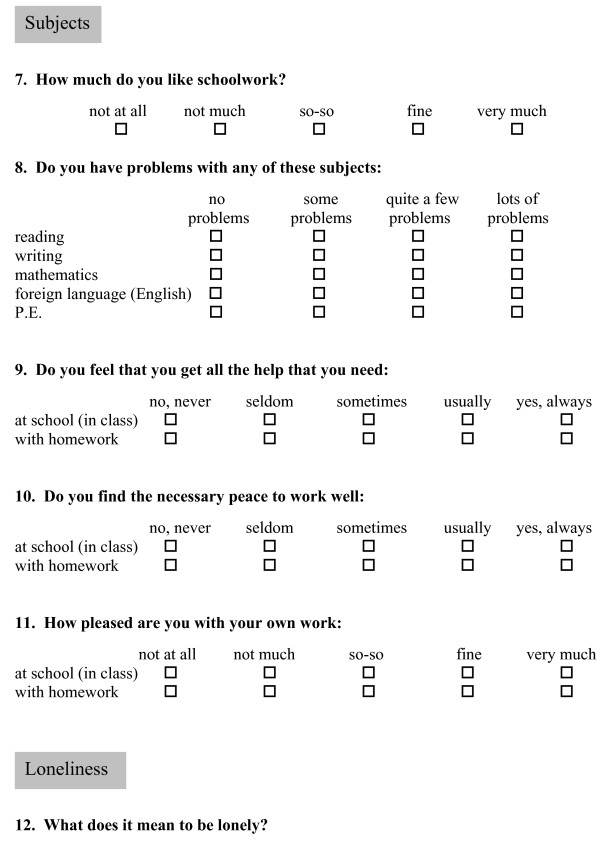
An example of questions from the *School wellbeing Student questionnaire*, developed by Audhild Løhre.

The questionnaire consisted of a combination of items that are assumed to promote health, and items that may be adversely associated with health. Factors assumed to adversely influence health included perceived academic problems, disturbances at work, being bothered in class, loneliness and victimization (being bullied). Among variables assumed to promote health were enjoyment in doing school work, a feeling of receiving help and assistance when needed, and satisfaction with performed school work. In addition, supportiveness of friends, peers and teachers was assumed to promote health. Responses to the questions were ranked on ordinal scales, with four or five response options (see Figure 1). The given responses should be relevant for the current school year. The assumed promoting and adverse factors have been described elsewhere [38].

### Ethics

The survey was approved by the statutory School Collaborative Committees, and the collection of data was approved by The Norwegian Data Inspectorate.

### Statistics

The analyses were performed with proportional odds logistic regression [39] using sadness, anxiety, stomach ache and headache as dependent variables. First, each factor was included separately as a covariate, adjusting only for gender and grade. Thereafter, all covariates were included simultaneously in a multivariable model. These analyses were also carried out separately for boys and girls.

The health symptoms were categorical ordinal variables with five levels, and applying proportional odds logistic regression is expected to be more efficient than using binary logistic regression [40,41]. In a binary logistic regression, the dependent variable had to be dichotomized at one of four alternative cut points. Proportional odds logistic regression is equivalent to performing four binary logistic regression analyses simultaneously, and the model assumes the odds ratio to be the same for every cut point.

All tests were two-sided, and p-values < 0.05 were considered significant. The statistical analyses were performed in SPSS for Windows (version 15.0 SPSS, Chicago, Illinois).

## Results

Among 419 participating children (230 boys and 189 girls), gender was evenly distributed by school grade [38]. Table 1 shows children's scores for the dependent variables; sadness, anxiety, stomach ache and headache, as well as for each independent, potentially explanatory factor. Most of the children (between 67% and 83%) reported never or seldom to have experienced any of the four symptoms, whereas about one in four children had experienced one or more of the symptoms now and then or more often. The score distribution for the independent variables was similar to the distribution of the outcomes, with the majority of children reporting the two best scores.

**Table 1:** Distribution of response options for dependent1 and independent2 variables

	Response options							
				
	1	2	3	4	5	Total		
Variables	%	%	%	%	%	N	Median	IQR*
Sadness^1a ^	24.5	48.9	23.5	2.7	0.5	413	2	2-3
Anxiety^1a^	54.7	28.0	12.9	3.2	1.2	411	1	1-2
Stomach ache^1a^	39.6	31.9	21.7	5.1	1.7	414	2	1-3
Headache^1a^	38.7	28.5	23.6	7.3	1.9	411	2	1-3
Academic problems^2b^	26.3	55.4	13.6	4.8		419	2	1-2
Disturbed work^2a^	19.2	39.3	29.5	9.4	2.6	417	2	2-3
Bothered in class^2a^	84.3	7.4	7.6	0.7	0	408	1	1-1
Loneliness^2a^	60.5	21.5	14.8	1.4	1.7	418	1	1-2
Victimization^2a^	55.2	24.2	16.5	2.2	1.9	417	1	1-2
School work enjoyment^2c^	2.6	4.8	48.4	35.6	8.6	419	3	3-4
Necessary academic help^2c^	1.0	3.4	11.8	43.2	40.6	414	4	4-5
School work satisfaction^2c^	1.4	3.3	32.5	46.7	16.0	418	4	3-4
Friends^2c^	0.2	2.6	15.8	19.4	62.0	418	5	4-5
Supportive peers^2d^	17.5	25.3	15.2	42.0		388	3	2-4
Supportive teacher^2d^	17.0	21.2	18.6	43.2		377	3	2-4

* 25-75th percentile

^a ^From 1 (best) to 5 (worst)

^b ^From 1 (best) to 4 (worst)

^c ^From 1 (worst) to 5 (best)

^d ^From 1 (worst) to 4 (best)

We assessed the association of each independent variable with the respective scores for sadness, anxiety, stomach ache and headache. The left part of Table 2, 3, 4, and 5 show the association of each independent variable, with adjustment for gender and grade. In the right part of the tables, the associations are also adjusted for the other variables listed in the table.

**Table 2:** Proportional odds logistic regression with sadness as dependent variable

	Each covariate adjusted only for gender and grade		All covariates, gender and grade, included in the model	
	
	Odds ratio		Odds ratio	
Covariates*	Estimate (95% CI)	p-value	Estimate (95% CI)	p-value
*Adverse factors*				
Academic problems	1.74 (1.35 to 2.23)	< 0.001	1.28 (0.94 to 1.74)	0.117
Disturbed work	1.31 (1.09 to 1.58)	0.005	0.98 (0.77 to 1.24)	0.838
Bothered during lessons	2.26 (1.64 to 3.10)	< 0.001	1.41 (0.96 to 2.08)	0.083
Loneliness	2.08 (1.67 to 2.59)	< 0.001	1.94 (1.42 to 2.64)	< 0.001
Victimization	1.56 (1.27 to 1.91)	< 0.001	0.93 (0.71 to 1.23)	0.613
*Promoting factors*				
School work enjoyment	0.82 (0.65 to 1.03)	0.095	0.97 (0.73 to 1.29)	0.837
Necessary academic help	0.58 (0.46 to 0.74)	< 0.001	0.78 (0.57 to 1.06)	0.115
School work satisfaction	0.73 (0.59 to 0.92)	0.007	0.94 (0.71 to 1.24)	0.651
Friends	0.66 (0.53 to 0.83)	< 0.001	0.82 (0.63 to 1.07)	0.141
Supportive peers	0.94 (0.80 to 1.11)	0.481	1.03 (0.85 to 1.24)	0.764
Supportive teacher	0.88 (0.73 to 1.06)	0.193	0.96 (0.77 to 1.19)	0.703

* Covariates are factors assumed to be associated with children's sadness

**Table 3:** Proportional odds logistic regression with anxiety as dependent variable

	Each covariate adjusted only for gender and grade		All covariates, gender and grade, included in the model	
	
	Odds ratio		Odds ratio	
Covariates*	Estimate (95% CI)	p-value	Estimate (95% CI)	p-value
*Adverse factors*				
Academic problems	2.22 (1.70 to 2.89)	< 0.001	1.59 (1.14 to 2.21)	0.006
Disturbed work	1.51 (1.24 to 1.83)	< 0.001	1.16 (0.90 to 1.50)	0.252
Bothered during lessons	2.49 (1.82 to 3.40)	< 0.001	1.54 (1.04 to 2.27)	0.032
Loneliness	2.31 (1.86 to 2.88)	< 0.001	1.78 (1.31 to 2.42)	< 0.001
Victimization	1.81 (1.47 to 2.22)	< 0.001	1.17 (0.88 to 1.56)	0.287
*Promoting factors*				
School work enjoyment	0.78 (0.61 to 1.00)	0.052	0.91 (0.67 to 1.23)	0.520
Necessary academic help	0.62 (0.49 to 0.79)	< 0.001	1.18 (0.85 to 1.64)	0.326
School work satisfaction	0.70 (0.55 to 0.89)	0.003	1.02 (0.76 to 1.38)	0.874
Friends	0.71 (0.57 to 0.88)	0.002	0.86 (0.65 to 1.14)	0.296
Supportive peers	1.05 (0.88 to 1.25)	0.580	1.09 (0.89 to 1.34)	0.388
Supportive teacher	0.95 (0.78 to 1.15)	0.573	1.01 (0.80 to 1.29)	0.904

* Covariates are factors assumed to be associated with children's anxiety

**Table 4:** Proportional odds logistic regression with stomach ache as dependent variable

	Each covariate adjusted only for gender and grade		All covariates, gender and grade, included in the model	
	
	Odds ratio		Odds ratio	
Covariates*	Estimate (95% CI)	p-value	Estimate (95% CI)	p-value
*Adverse factors*				
Academic problems	1.45 (1.14 to 1.85)	0.003	1.08 (0.80 to 1.46)	0.629
Disturbed work	1.22 (1.02 to 1.47)	0.032	1.12 (0.89 to 1.42)	0.336
Bothered during lessons	1.80 (1.33 to 2.44)	< 0.001	1.44 (0.99 to 2.09)	0.057
Loneliness	1.65 (1.35 to 2.03)	< 0.001	1.33 (0.99 to 1.78)	0.056
Victimization	1.52 (1.25 to 1.85)	< 0.001	1.09 (0.83 to 1.42)	0.538
*Promoting factors*				
School work enjoyment	0.96 (0.76 to 1.20)	0.698	0.99 (0.75 to 1.31)	0.964
Necessary academic help	0.68 (0.54 to 0.86)	0.001	0.74 (0.54 to 1.01)	0.055
School work satisfaction	0.89 (0.72 to 1.11)	0.307	1.10 (0.83 to 1.44)	0.509
Friends	0.82 (0.66 to 1.01)	0.063	1.04 (0.80 to 1.35)	0.775
Supportive peers	1.09 (0.93 to 1.29)	0.297	1.07 (0.89 to 1.30)	0.451
Supportive teacher	1.02 (0.85 to 1.23)	0.825	1.15 (0.92 to 1.43)	0.227

* Covariates are factors assumed to be associated with children's stomach ache

**Table 5:** Proportional odds logistic regression with headache as dependent variable

	Each covariate adjusted only for gender and grade		All covariates, gender and grade, included in the model	
	
	Odds ratio		Odds ratio	
Covariates*	Estimate (95% CI)	p-value	Estimate (95% CI)	p-value
*Adverse factors*				
Academic problems	1.44 (1.13 to 1.84)	0.003	1.10 (0.81 to 1.48)	0.542
Disturbed work	1.43 (1.19 to 1.73)	< 0.001	1.24 (0.98 to 1.57)	0.071
Bothered during lessons	1.90 (1.41 to 2.58)	< 0.001	1.28 (0.88 to 1.85)	0.198
Loneliness	1.61 (1.32 to 1.98)	< 0.001	1.47 (1.10 to 1.96)	0.010
Victimization	1.57 (1.29 to 1.91)	< 0.001	1.10 (0.84 to 1.44)	0.486
*Promoting factors*				
School work enjoyment	0.82 (0.65 to 1.03)	0.085	0.99 (0.75 to 1.30)	0.917
Necessary academic help	0.67 (0.53 to 0.85)	0.001	0.93 (0.69 to 1.27)	0.658
School work satisfaction	0.75 (0.60 to 0.93)	0.009	0.89 (0.68 to 1.17)	0.418
Friends	0.78 (0.63 to 0.96)	0.022	0.95 (0.74 to 1.24)	0.728
Supportive peers	1.04 (0.89 to 1.23)	0.608	1.12 (0.93 to 1.34)	0.251
Supportive teacher	0.84 (0.70 to 1.00)	0.056	0.92 (0.74 to 1.14)	0.452

* Covariates are factors assumed to be associated with children's headache

### Sadness

In the analyses only adjusting for gender and grade (left part of Table 2), most of the variables were significantly associated with sadness scores in the expected direction. Thus, all variables indicating problems in lessons or recess were related to higher degree of sadness, whereas experiencing necessary academic help, perceived satisfaction with the school work, and having many friends were associated with lower sadness scores. In the multivariable analysis (right part of Table 2), most of the associations were attenuated, and "loneliness" was the only variable that remained strongly associated with sadness (odds ratio, 1.94, 95% CI 1.42 to 2.64).

In separate analyses of boys and girls (results not tabulated), the results were similar for both genders, and "loneliness" was the only significant contributor to sadness after multivariable adjustment.

### Anxiety

The results related to anxiety (left part of Table 3) correspond to the findings for sadness. However, after multivariable adjustment (right part of Table 3), three variables remained as possible contributors to the anxiety scores. Thus, experiencing academic problems (odds ratio, 1.59, 95% CI 1.14 to 2.21), being bothered during lessons (odds ratio, 1.54, 95% CI 1.04 to 2.27) and loneliness (odds ratio, 1.78, 95% CI 1.31 to 2.42) were all associated with higher degree of anxiety in the multivariable analysis.

Separate analyses by gender showed that experiencing academic problems was the only variable associated with anxiety among boys (odds ratio, 1.69, 95% CI 1.04 to 2.74), whereas in girls, being bothered during lessons (odds ratio, 1.80, 95% CI 1.03 to 3.14) and loneliness (odds ratio, 2.53, 95% CI 1.58 to 4.06) were strongly associated with anxiety.

### Stomach ache

All the assumed adverse factors were associated with higher degree of stomach ache (left part of Table 4), whereas receiving necessary academic help was associated with a low degree of stomach ache. After multivariable adjustment (right part of Table 4), most of these associations were fully attenuated, but associations related to being bothered during lessons, loneliness and receiving necessary academic help remained of borderline statistical significance.

In separate analyses by gender, there were no clear associations with stomach ache among boys. For girls, however, receiving necessary academic help was negatively associated with the reported prevalence (odds ratio, 0.51, 95% CI 0.30 to 0.87).

### Headache

The initial results for headache correspond to the patterns observed for sadness and anxiety (left part of Table 5), but after multivariable adjustment, loneliness (odds ratio, 1.47, 95% CI 1.10 to 1.96) was the only variable that remained statistically significant, suggesting that loneliness is associated with a higher prevalence of headache (right part of Table 5).

In separate analyses by gender, no clear associations with headache were present for boys, but among girls, being disturbed in school work (odds ratio, 1.79, 95% CI 1.21 to 2.65) and loneliness (odds ratio, 1.66, 95% CI 1.08 to 2.57) were both strongly and positively associated with the prevalence of headache.

## Discussion

In this cross-sectional study of self-reported internalizing and somatic symptoms among more than 400 school children, we found that perceived loneliness was strongly associated with the prevalence of sadness, anxiety and headache, also after adjustment for a number of potentially confounding factors. In separate analyses of boys and girls, loneliness in boys was strongly associated with sadness, whereas in girls, the association of loneliness was equally strong for sadness, anxiety and headache.

The associations of loneliness were robust, and did not substantially change from the crude (only adjusting for grade and gender) to the multivariable analysis. The results suggest that loneliness may be particularly important among girls, since loneliness was the most important correlate to high scores for three of the four symptoms.

The study was conducted in public schools in rural communities, ranging from inland to coastal environments. The population base and the very high attendance are strengths of the study, but it is a weakness that children from urban settings were not included. In the data collection, younger children were interviewed by school nurses, whereas older children completed the questionnaire themselves. Although the nurses were trained for this task, the possibility that the different procedures could have influenced the responders and introduced systematic differences in results between younger and older children can not be excluded.

Also, the cross-sectional design is a limitation of this study. That the children simultaneously reported exposures and outcomes may lead to inter-related responses to the questions, and could have caused stronger associations between explanatory factors and health outcomes. Thus, collecting outcomes at a later stage could have yielded different results. Therefore, the findings should be interpreted with caution, since cross sectional designs limit the possibility to study causal effects.

The internalizing and somatic symptoms that we used as outcome measures in this study are common, and there is evidence suggesting that self-reports of emotional and somatic symptoms are reasonably reliable in studies of health in adolescence [42]. Internalizing and somatic symptoms may infer with children's daily living and cause absence from school [1]. Further, previous studies of internalizing or somatic symptoms in childhood and adolescence have shown an increased risk of anxiety disorders, depression, and somatic illness later in life [3,4,9,12,14,23,24].

In the initial analyses (only adjusting for gender and grade) among factors that were assumed to promote health, children's satisfaction with academic work and the help they receive from teachers were associated with a relatively lower prevalence of symptoms. After mutual adjustment for other variables, only the negative association of help from teachers with stomach ache in girls remained significant. Previously, it has been suggested that academic satisfaction may be beneficial for children's health [43], and that support from teachers may provide protection against poor health [35,44].

Each factor that was assumed to be adversely related to health was associated with higher scores for each of the four symtoms in the crude analyses, but after mutual adjustment for other potentially explanatory variables, most of the initial associations were fully attenuated. In other studies, multivariable adjustment also attenuated the estimates, but to different degrees [42,45-47]. Victimization caused by bullying is an example of a factor that has shown robust associations, also in multivariable analyses.

In this study, loneliness was the only factor that retained the strong relation to poorer health after adjustment for other potentially confounding factors. We cannot rule out the possibility that factors that we failed to include in the study, at least in part, may explain the associations of loneliness. Thus, it has been suggested that close friendship and peer acceptance could modify effects related to loneliness [48-50]. On the other hand, it may be equally plausible that the variable loneliness captures something that in itself is strongly associated with the internalizing and somatic symptoms that we have studied. Sadness may be a key emotion for both depression [6,51,52] and loneliness [48], but the link of loneliness to the physical complaints, headache and stomach pain, may not be easily explained, unless these complaints represent somatic expressions of underlying emotional distress [5,7,53].

Only a few studies have assessed the association of perceived loneliness with health problems in childhood and adolescence, and to our knowledge, no previous study has assessed loneliness in relation to headache or stomach pain. Nonetheless, the strong associations that we found for loneliness and emotional distress are in line with previous findings. In cross-sectional studies, it has been suggested that loneliness is associated with both anxiety [54,55] and depression [30,56], and that persistent loneliness may contribute to later emotional disorders [56]. From a recent prospective study that followed children from childhood to adolescence, it was reported that measures of loneliness at the age of 5 and 9 years could predict depressive symptoms at 13 years of age [57].

Few studies have compared internalizing or somatic symptoms between girls and boys in relation to loneliness, and there are no consistent gender differences [56]. We found, however, a strong association of loneliness with anxiety and headache among adolescent girls, but not in boys, whereas for sadness, there was a clear association of loneliness for both genders.

## Conclusions

In this population study of children between 7 and 16 years of age, perceived loneliness appears to be of special importance in relation to internalizing and somatic symptoms, and for girls, perceived loneliness may be particularly important in relation to emotional distress (sadness and anxiety) and physical complaints (headache). Longitudinal studies that measure the impact of factors that are associated with perceived loneliness and their relation with subsequent health problems are recommended.

Emotional and somatic symptoms are common in childhood and adolescence. Teachers, school nurses, clinicians, and others need to be aware of the strong relation between loneliness and ill health, and daily routines should be established to reduce loneliness among school children. It is possible that a caring attention from teachers and school nurses combined with strategic planning of activities and peer collaboration may reduce loneliness among the children.

## Competing interests

The authors declare that they have no competing interests.

## Authors' contributions

The present cross-sectional study is part of a two year follow-up, planned and administered by AL. All the three authors participated in designing the study. AL and SL did the analyses. AL, SL, and LJV interpreted the data and wrote the paper. All authors read and approved the final manuscript.
